# Low levels of estradiol are associated with elevated conditioned responding during fear extinction and with intrusive memories in daily life

**DOI:** 10.1016/j.nlm.2014.10.001

**Published:** 2014-12

**Authors:** Melanie Wegerer, Hubert Kerschbaum, Jens Blechert, Frank H. Wilhelm

**Affiliations:** a Division of Clinical Psychology, Psychotherapy, and Health Psychology, Department of Psychology, University of Salzburg, Hellbrunnerstraße 34, 5020 Salzburg, Austria; b Department of Cell Biology, University of Salzburg, Hellbrunnerstraße 34, 5020 Salzburg, Austria

**Keywords:** Classical conditioning, Trauma film, Intrusions, Anxiety disorders, Estrogen, Menstrual cycle

## Abstract

Posttraumatic stress disorder (PTSD) can be conceptualized as a disorder of emotional memory showing strong (conditioned) responses to trauma reminders and intrusive memories among other symptoms. Women are at greater risk of developing PTSD than men. Recent studies have demonstrated an influence of ovarian steroid hormones in both fear conditioning and intrusive memory paradigms. However, although intrusive memories are considered non-extinguished emotional reactions to trauma reminders, none of the previous studies has investigated effects of ovarian hormones on fear conditioning mechanisms and intrusive memories in conjunction. This may have contributed to an overall inconsistent picture of the role of these hormones in emotional learning and memory. To remedy this, we exposed 37 healthy women with a natural menstrual cycle (during early follicular or luteal cycle phase) to a novel conditioned-intrusion paradigm designed to model real-life traumatic experiences. The paradigm included a differential fear conditioning procedure with short violent film clips as unconditioned stimuli. Intrusive memories about the film clips were assessed ambulatorily on subsequent days. Women with lower levels of estradiol displayed elevated differential conditioned skin conductance responding during fear extinction and showed stronger intrusive memories. The inverse relationship between estradiol and intrusive memories was at least partially accounted for by the conditioned responding observed during fear extinction. Progesterone levels were not associated with either fear acquisition/extinction or with intrusive memories. This suggests that lower levels of estradiol might promote stronger symptoms of PTSD through associative processes.

## Highlights

• Intrusive memories can be seen as conditioned responses to trauma reminders.
• Novel conditioned-intrusion paradigm models both fear conditioning and intrusions.
• Low estradiol is related to higher conditioned responses during fear extinction.
• Low estradiol is related to higher intrusive memory strength in daily life.
• Conditioned responding during extinction partially explains the latter relationship.

## 1 Introduction

Posttraumatic stress disorder (PTSD) and other anxiety disorders are highly disabling conditions, with women being at greater risk of PTSD than men (Olff et al., 2007, Tolin and Foa, 2006). Contemporary cognitive-behavioral theories assume that PTSD constitutes a disorder of emotional memory with strong responding to trauma reminders and intrusive memories belonging to its most prominent symptoms (Ehlers and Clark, 2000, Mineka and Oehlberg, 2008, VanElzakker et al., 2014). According to the fear conditioning approach, a traumatic event can be seen as an unconditioned stimulus (UCS) that elicits an unconditioned response (UCR) characterized by strong arousal and fear. Co-occurrence with the traumatic event turns stimuli such as sounds, sights or smells into conditioned stimuli (CSs), signalling impending danger and eliciting a conditioned fear response (CR) even when being presented alone later. Despite sometimes being experienced as ‘coming out of the blue’, intrusive memories are frequently triggered by such CSs. Based on this account, intrusive memories can be regarded as conditioned emotional reactions to trauma reminders (Foa et al., 1992, Mineka and Oehlberg, 2008). Ehlers and Clark (2000) suggested that associative learning is particularly strong in patients with PTSD, thus making the triggering of intrusive memories even more likely. Similarly, Orr et al. (2000) proposed that the propensity to acquire larger as well as more persistent autonomic responses to an aversive CS, i.e. stronger fear acquisition and/or weaker extinction (higher fear conditionability), might render individuals more likely to develop symptoms of PTSD. This notion was supported by findings of heightened conditioned responding during acquisition or extinction in patients with PTSD (Blechert et al., 2007, Lommen et al., 2013, Norrholm et al., 2011, Orr et al., 2000, Peri et al., 2000, Wessa and Flor, 2007).

Owing to the higher risk for PTSD in women, research has recently begun to study fear acquisition and extinction (e.g. Glover et al., 2012, Zeidan et al., 2011) as well as intrusive memories (e.g. Bryant et al., 2011, Ferree et al., 2011) specifically in women and in relation to the ovarian steroid hormones estradiol and progesterone. Localization of estradiol and progesterone receptors is not restricted to the hypothalamus, but receptors have also been identified in a multitude of other brain areas, including the hippocampus, amygdala, and prefrontal cortex (Brinton et al., 2008, Cui et al., 2013, McEwen et al., 2012, Montague et al., 2008). Accordingly, estradiol and progesterone are not only involved in the control of reproductive physiology and behavior, but have been found to more broadly modulate cognitive as well as emotional processes (Farage et al., 2008, Lebron-Milad and Milad, 2012, van Wingen et al., 2011). The latter is, for example, illustrated by symptoms of anxiety and depression being particularly common during periods with extreme changes in ovarian hormone levels, such as during postpartum and perimenopausal periods (Altshuler et al., 1998, Moses-Kolko et al., 2009, Schmidt and Rubinow, 2009). However, effects of ovarian hormones on cognitive-emotional processing have also become evident on the basis of more subtle fluctuations of these hormones as they occur during the menstrual cycle (Sacher, Okon-Singer, & Villringer, 2013). Estradiol levels are low during the early follicular cycle phase, peak before ovulation in the late follicular phase and decrease to a moderate level during the luteal phase, whereas progesterone levels are low during the follicular phase and peak at the mid-luteal phase (Sacher et al., 2013).

Crucially, with respect to potential relationships between ovarian hormones and cognitive-emotional processing as revealed during *fear conditioning*, an initial study by Milad et al. (2010) reported that naturally cycling women with low as compared to high levels of estradiol exhibited reduced extinction recall (i.e. more fear in an extinction recall session that was conducted one day after initial fear acquisition and extinction training) (but see also Milad et al., 2006). Zeidan et al. (2011) replicated this finding and additionally found higher activation of brain regions such as the ventromedial prefrontal cortex during extinction recall but also during initial extinction learning in women with higher levels of estradiol. Furthermore, it has been shown that the use of hormonal contraceptives – which are known to decrease ovarian production of estradiol and progesterone – impairs extinction recall and that, on the other hand, administration of estradiol has the potential to enhance women’s ability to recall extinction memory (Graham & Milad, 2013). Importantly, a role of estradiol in fear extinction is not only supported by studies in healthy participants, but has also been demonstrated in traumatized women (Glover et al., 2012). Furthermore, Glover et al. (2012) found average PTSD symptoms to be higher in traumatized women with low as compared to high estradiol levels. In summary, studies reported above agree on an association between estradiol and fear extinction processes and have led to the conclusion that low levels of estradiol might constitute a vulnerability factor for symptoms of PTSD (respective findings are reviewed by Lebron-Milad, Graham, & Milad, 2012, and Hiroi & Neumaier, 2011).

A separate line of research has investigated associations between estradiol, progesterone, or menstrual cycle phase and *intrusive memories*. Ferree and Cahill (2009) found that women in the luteal phase reported more spontaneous intrusive memories of film clips than women in the follicular phase. Similarly, Bryant et al. (2011) reported that traumatized women were more likely to experience flashback memories if they were in the luteal phase either at the time of trauma or at the time of assessment. However, neither of the two studies assessed ovarian hormone concentrations. This gap was closed by Ferree et al. (2011) observing a positive correlation between progesterone levels and spontaneous intrusive memories that women reported following the viewing of emotional films. More recently, Cheung, Chervonsky, Felmingham, and Bryant (2013) failed to replicate the latter finding. They found that levels of estradiol rather than progesterone were positively related to intrusive memories of negative images. However, it should be noted that Cheung et al. (2013) neither excluded women on hormonal contraceptives nor assessed women’s menstrual cycle phase, which could potentially account for the different study outcomes. Finally, a recent study by Soni, Curran, and Kamboj (2013) found a negative relationship between intrusive memories and the estradiol-to-progesterone ratio rather than observing correlations with either estradiol or progesterone alone.

Thus, compared to the emerging picture in fear conditioning research, the intrusive memory literature remains inconsistent. Importantly, despite the theoretical considerations that fear conditioning and intrusive memories are functionally connected (see above), none of the reviewed studies has investigated the two processes in conjunction, thereby possibly contributing to the discrepant findings between these two lines of research. It is likely that methodological differences between these two paradigms precluded their integrated study: Fear conditioning studies mostly rely on UCSs such as electrical stimulation (e.g. Milad et al., 2006, Milad et al., 2010) or air blasts (Glover et al., 2012) that only partially depict the typical features (e.g. dynamic, multimodal events) of situations involved in fear learning in real life. Such UCSs are unlikely to generate the kind of complex memories that could later give rise to intrusive memories and are thus inappropriate to investigate fear conditioning and intrusive memories in conjunction. To overcome this problem, we developed a *conditioned-intrusion paradigm* to model the fear learning process in a more naturalistic manner: Short aversive film clips depicting severe violence serve as UCS and are predicted by neutral sound clips as CSs. Using the paradigm, reliable differential fear conditioning as well as intrusive memories on subsequent days were obtained. Importantly, residual conditioned responding during extinction (indicating both stronger and more persistent conditioned responding, hence higher ‘fear conditionability’ as defined by Orr et al., 2000) was positively correlated with subsequent intrusive memory strength, thereby encouraging further investigation of intrusive memories within a fear conditioning framework (Wegerer, Blechert, Kerschbaum, & Wilhelm, 2013).

The current study extends this work toward investigating the role of estradiol and progesterone in fear conditioning and intrusive memories, studied together in the conditioned-intrusion paradigm. Lower levels of estradiol were expected to be accompanied by stronger conditioned responding during fear extinction (Glover et al., 2012, Zeidan et al., 2011), whereas acquisition response levels should be unaffected. Furthermore, inasmuch as intrusive memories represent non-extinguished conditioned responding to trauma reminders, low estradiol should also be associated with higher intrusive memory strength and this link should be mediated by the degree of conditioned responding observed during fear extinction. For progesterone predictions were less clear since the reviewed evidence failed to document an effect on fear conditioning. Similarly, predictions for intrusive memories are complicated by inconsistent findings (see above; Cheung et al., 2013, Ferree et al., 2011, Soni et al., 2013) and by the fact that some studies did not assess ovarian hormone levels but analyzed data only with respect to self-reported menstrual cycle phase (Bryant et al., 2011, Ferree and Cahill, 2009). Yet, a related line of research, focusing on deliberate (rather than spontaneous, intrusive) emotional memory recall, found progesterone levels to predict recall and recognition memory for negative images (Ertman et al., 2011, Felmingham et al., 2012). Together with the initial evidence for an association between progesterone and intrusive memories (see above; Ferree et al., 2011) these latter studies point to a more general effect of progesterone on enhancing aversive (declarative) memory. Based on this background, we made the more tentative prediction that progesterone levels are positively associated with intrusive memories (in the absence of an association with – or mediation by – conditioned responding during extinction).

## 2 Methods

### 2.1 Participants

Sixty-six healthy women aged 18–35 years were recruited at the University of Salzburg, Austria, and participated in exchange for course credits or 25 euros. Participants did not report any current mental or neurological disorders and were free of any medication. Furthermore, none of the participants reported past experiences of severe interpersonal violence. In an earlier report we have described data of this sample with regard to the general relationship between fear conditioning processes and intrusive memories. Effects of ovarian hormones were, however, not covered by that report (Wegerer, Blechert, Kerschbaum, et al., 2013). To be included in the current analyses, women had to be free of hormonal contraceptives for at least three months to preclude any influences of external hormonal administration. Furthermore, they had to report regular menstrual cycles and no diagnosis of premenstrual dysphoric disorder. In total, 20 participants had to be excluded based on these criteria. Assessments were scheduled to take place either during the women’s early follicular phase (day 1–7 of menstrual cycle) or the luteal phase (7 ± 5 days before onset of next menstruation). This was done to sample a broad range of individual hormonal concentrations while at the same time allowing explorative comparisons of different menstrual cycle phases (see Appendix A). The current cycle phase was assessed by asking participants about the first day of their last menses. Additionally, onset of the next menses was evaluated at follow-up to confirm cycle phases. Participants who happened to be tested outside of the respective time windows were excluded from further analyses since a clear assignment of these women to cycle phases was not possible (four participants).^1^ Further exclusion criteria were early termination of the study (two participants), excessive consumption exceeding three times per week of films or video games including severe violence (one participant), and insufficient unconditioned responding (criteria explained below; two participants), thus reducing the final sample to 37 participants. The ethics committee of the University of Salzburg approved the study and written informed consent was obtained from all participants according to the Declaration of Helsinki. Before participation, experimental procedures were described in detail, including the presentation of exemplary movie scenes resembling those shown within the study. Participants were furthermore informed that they could indicate to stop the experiment and withdraw from their participation at any time with full compensation.

### 2.2 Procedure

Since the full experimental protocol has been explained elsewhere (Wegerer, Blechert, Kerschbaum, et al., 2013, Wegerer, Blechert, Wilhelm, et al., 2013), we describe here in detail only procedures that are of particular interest for the current analyses. Laboratory testing started between 8:30 and 15:00 for all participants.^2^ After being welcomed to the laboratory and signing the informed consent, participants completed several questionnaires, including an assessment of trait anxiety (State–Trait Anxiety Inventory, STAI, German version by Laux, Glanzmann, Schaffner, & Spielberger, 1981) and depressive symptoms (General Depression Scale, ADS–L; German version by Hautzinger & Bailer, 1993). Furthermore, general medical and psychological health conditions (including menstrual cycle characteristics and potential past or current usage of hormonal contraception) as well as consumption of TV and film footage or video games depicting severe violence were assessed.^3^ Next, participants were asked to gently flush out their mouth with some water as a preparation for the subsequent drawing of the saliva sample. Participants were then seated in front of a computer monitor where electrodes for skin conductance measurement were attached. Thereafter, the saliva sample was collected (i.e. 10 min after mouth flushing) and subsequently frozen (see below for details). Next, participants sat quietly for 2.5 min to allow adaptation to the laboratory and to record their baseline skin conductance level (SCL). Subsequently, the fear conditioning procedure commenced.

#### 2.2.1 Fear conditioning procedure

Two naturalistic sounds (sound A: sound of a typewriter; sound B: clock ticking; each with a duration of 5 s) had been selected based on data of a pilot study to serve as conditioned stimuli (CSs). Furthermore, three film clips (each with a duration of 25 s), depicting severe violence between two persons or the moment immediately after such an attack showing a severely injured person, were selected to be used as UCS. Data of a pilot study had confirmed that the three film clips were matched on both negative valence as well as high arousal (Wegerer, Blechert, Kerschbaum, et al., 2013, Wegerer, Blechert, Wilhelm, et al., 2013). CS sounds were assigned to CS^+^ (i.e. sound that was later followed by a film clip) and CS^−^ (i.e. sound that was never followed by a film clip), counterbalanced across the participants. The conditioning procedure consisted of a habituation, an acquisition, and an extinction phase. Prior to habituation, written instructions informed participants that they were going to hear the sound of a typewriter and of a clock and that there were no aversive film clips going to be displayed during this phase of the experiment. During the habituation phase, CS^+^ and CS^−^ sounds were presented six times for durations of 5 s each. The order of CS^+^ and CS^−^ stimuli was determined pseudorandomly for the whole task, so that no more than two stimuli of the same type (CS^+^, CS^−^) would be presented in a row. Throughout the task, an inter-trial interval of 12–20 s was used (order of inter-trial intervals with different length again determined pseudorandomly to prevent succession of several ITIs with same length). After the habituation phase, participants were informed that the two sounds would be presented again in the next part of the experiment and that one of the sounds could now be followed by a film scene, whereas the other sound would not be followed by film scenes (Blechert et al., 2007, Michael et al., 2007). The acquisition phase consisted of eight CS^+^ sounds of 5 s duration, of which six were followed by an aversive film clip (each of three film clips presented two times in pseudorandom order, 75% reinforcement). During the film clips the CS^+^ sound kept playing at a lowered volume in the background. The acquisition phase further comprised six CS^−^ sound presentations of 5 s duration not followed by a film clip. During the extinction phase, the CS^+^ and CS^−^ were both presented six times with the CS^+^ sound no longer followed by a film clip. At the end of the whole fear conditioning procedure, participants rated valence separately for each film clip (visual analog scale; 0 = very pleasant, 100 = very unpleasant).

#### 2.2.2 Ambulatory assessment of intrusive memories

At the end of the laboratory study, participants were informed that they would be sent a link for an Internet questionnaire on the following three evenings (i.e. on the day of the experiment and the two following days), which they were requested to complete shortly before going to bed on each day. The questionnaire constituted an adapted version of the Intrusion Memory Questionnaire (IMQ; Ehring et al., 2009, Zetsche et al., 2009) and assessed involuntary retrieval of intrusive memories in daily life. The IMQ was adapted to assess the number and duration of memories (in minutes) as well as distress (visual analog scale with the anchors ‘0 = not distressing at all’ and ‘100 = extremely distressing’) elicited by memories experienced during the respective day. Note that we assessed intrusive memories for each day retrospectively on the respective evening rather than collecting continuous electronic diary data, since theoretical considerations (Wilhelm & Grossman, 2010) and a pilot study had indicated strong reactivity effects when participants were asked to record each intrusive memory immediately after its occurrence. To ensure the capturing of memories that most closely resembled intrusions in PTSD, participants were instructed on the kind of memories that they should report on at the outset of the questionnaire: *“We are interested in memories of the violent film clips that you experienced today. These can involve images, sounds, or thoughts about things that have been shown, but also thoughts or feelings that you had when watching the film clips. Please do not report on memories that you recalled deliberately (e.g. when telling someone else about the film scenes), but only on memories that came up spontaneously”* (Ehring et al., 2009, Zetsche et al., 2009). Note that the three items of the IMQ (number, duration, and distress of intrusive memories; see footnote^4^ for exact wording) represent meaningful and distinct information relating to intrusive memory strength in daily life (i.e. higher experienced intrusive memory strength can be due to a higher number, a higher duration, or higher distress caused by intrusive memories). To reduce alpha inflation in subsequent correlation analyses (see below) and to obtain a more reliable score of intrusive memories, we calculated an index of intrusive memory strength by building a sum score for the IMQ questionnaire for each day by standardizing and summing single items. (Single item responses were scaled differently and thus needed to be transformed into *z*-scores taking all subjects and points of measurement into account. For purposes of better illustration within figures, *z*-scores were further transformed into *t*-scores. However, means and standard deviations of raw values for number, duration, and distress of intrusive memories per day are reported in Table 1.) Cronbach alphas ranged between .706 < *α*<.842 for days 0 to 2, pointing to the feasibility of summing single items. Furthermore, sum scores were averaged over days to get a more reliable score of mean intrusive memory strength during everyday life; Cronbach alpha: .831. Finally, participants were orally debriefed and reimbursed for their study participation. Particular emphasis was given to the possibility of contacting the experimenters in case of further questions or distress due to the experiment.

**Table 1:** Means (and *SD*) for the three Intrusion Memory Questionnaire (IMQ) items during ambulatory assessments following the conditioning procedure.

	Intrusive memories		
	Day 0	Day 1	Day 2
	*M* (SD)	*M* (SD)	*M* (SD)
IMQ			
Number	4.76 (6.31)	2.62 (4.51)	1.60 (2.76)
Total duration (in min)	12.81 (17.65)	6.03 (9.17)	2.47 (3.59)
Distress (0–100)^a^	35.41 (29.59)	21.08 (26.33)	13.24 (22.37)

a 0 = not distressing at all, 100 = extremely distressing.

### 2.3 Salivary hormone assessment

In general, measuring estradiol and progesterone in saliva is considered to provide an accurate representation of the bioavailable fraction of these hormones in women of reproductive age (Bellem, Meiyappan, Romans, & Einstein, 2011). Saliva samples were taken by direct expectoration into a sterile tube since cotton Salivette-based methods may distort estradiol and progesterone measurements (Shirtcliff, Granger, Schwartz, & Curran, 2001). Participants had been instructed to refrain from drinking alcohol in the 24 h before participating in the laboratory study and to not eat, drink, or smoke 15 min before getting to the laboratory (corresponding to 45 min before the drawing of the saliva sample) to limit the confounding of hormonal assessments. After collection, saliva samples were frozen at −20 °C. Estradiol and progesterone levels were assessed using Demeditec Diagnostics (Kiel, Germany) ELISA kits and measured optically with a Lucy 2 Microplate Luminometer (Anthos Labtec Instruments GmbH, Wals, Austria) according to the manufacturer’s guidelines. For estradiol and progesterone, the kits have a detection range of 0.8–93 pg/mL and 0–5000 pg/mL and a sensitivity of 0.4 pg/mL and 5 pg/mL, respectively. Inter- and intra-assay variability are 2.1–4.3% and 2.6–6.9%, respectively, for estradiol and 8.6–10.1% and 6.0–9.6% for progesterone. Each saliva sample was divided into two replicates and levels of estradiol and progesterone were assessed separately in each. Values of both replicates were averaged to increase the reliability of hormone concentration estimates.

### 2.4 Apparatus and physiological recordings

E-Prime 2.0 was used to control stimulus presentation and behavioral data acquisition (Psychology Software Tools, Inc., Pittsburgh, PA, USA). Acoustic stimuli were presented via shielded earphones at a constant volume across participants. Skin conductance (SC) was measured using Ag/AgCl electrodes filled with isotonic electrode paste (Boucsein et al., 2012). Electrodes were placed on the middle phalanx of the index and middle fingers of the non-dominant hand. Recording of SC data was performed with a sampling rate of 1000 Hz using the software Polybench 1.22 (TMSi, Twente Medical Systems International, EJ Oldenzaal, Netherlands), a Porti 32-channels-amplifier (TMSi), and an SC-amplifier (Becker Meditec, Karlsruhe, Germany). Further analysis of SC data was conducted using ANSLAB 2.51 (Wilhelm & Peyk, 2005). Note that we refer to skin conductance in general as SC, whereas SCL specifically means skin conductance level (i.e. the tonic level of skin conductance) and SCR specifically indicates skin conductance response (i.e. phasic alterations in skin conductance elicited by stimuli) (Boucsein, 2012).

### 2.5 Data reduction and statistical analyses

Since our primary interest was to assess influences of estradiol and progesterone, continuous levels of these hormones were used in the main analyses. This can be considered more informative when aiming at characterizing the effects of specific hormones than comparing different menstrual cycle phases (Ferree et al., 2011, Glover et al., 2013). Nevertheless, explorative analyses comparing women during the early follicular and luteal cycle phase – which aimed at exploring differences between different, more complex hormonal milieus – are reported in Appendix A*.* To first check for possible relationships between estradiol and progesterone levels with demographic variables, psychometric characteristics, physiological baseline activation, or reactions to unconditioned stimuli, hormonal levels were correlated with the respective variables. For these and all following correlations, Spearman correlation coefficients (Spearman’s rho) were calculated in cases where variable pairs displayed deviation from bivariate normal distribution or were measured on an ordinal rather than an interval scale (Pearson correlation coefficients are indicated by *r*, Spearman correlation coefficients *rho* are indicated by *ρ* throughout the text).

Importantly, participants with a deficient unconditioned reaction (UCR) during fear conditioning were excluded for all analyses, since this could either imply measurement problems or insufficient aversiveness of film clips. A mean UCR <0.2 μS was used as exclusion criterion with UCR calculated by subtracting mean baseline SCL (−1 to 1 s relative to film clip onset, taking a SCR onset delay into account; Boucsein, 2012) from the maximum SCL during the remaining 24 s of the film clip, considering only the first presentation of each film clip. Two participants were excluded based on the UCR exclusion criterion. For all other participants and analyses, UCRs were normalized using the natural logarithm of 1 + UCR (in μS) and averaged over all film clips to obtain an indicator of individual mean unconditioned responding. For the conditioning procedure, we calculated a skin conductance response (SCR) by subtracting the average pre-CS baseline SCL (−2 to 0 s relative to CS onset) from the maximum CS SCL (0–6 s relative to CS onset, again taking an SCR response delay into account) and transformed SCR data using the natural logarithm of 1 + SCR (in μS). Responses to each CS-type were then averaged for each conditioning phase (see ANOVAs (Section 2.5.1.) and mediational analyses (Section 2.5.3.) below) or for three consecutive presentations resulting in two blocks per conditioning phase (see ANCOVAs (Section 2.5.1.) below).

#### 2.5.1 Statistical analyses of fear conditioning task

First, to test whether the fear conditioning procedure has evoked significant fear learning, a repeated-measures ANOVA was calculated for SCRs including CS-type (CS^+^, CS^−^) and Conditioning phase (habituation, acquisition, extinction) as within-subject factors. For acquisition, only CS^+^ presentations that were reinforced by an aversive film-UCS were included in this and the following analyses (all results remained unchanged when including unreinforced CS^+^ in the respective analyses). Second, we investigated the influences of estradiol and progesterone on differential conditioned SCRs by calculating a repeated-measure ANCOVA for habituation, acquisition, and extinction, respectively. The outcome variable ‘differential SCR’ was calculated by subtracting SCRs to CS^−^ from SCRs to CS^+^ for the respective time period. For each conditioning phase, this ANCOVA included Time (first and second block of the respective conditioning phase) as a within-subject factor and estradiol and progesterone levels as covariates. Note that a significant hormone main effect would indicate a significant relationship between that hormone and differential SCR in the respective conditioning phase.^5^ Even though a significant hormone effect was only expected for the extinction phase (see above), results of habituation and acquisition are still reported to give a complete overview of the conditioning task. When the sphericity assumption was violated, the Greenhouse–Geisser correction for repeated measures was applied with *ε* and nominal degrees of freedom being reported. Significant main or interaction effects were further explored using *t*-tests. Additional ANCOVAs explored whether UCR strength or trait anxiety and depressive symptoms modulated the results of the above analyses.

#### 2.5.2 Statistical analyses of intrusive memories

Correlations were calculated between hormonal concentrations and the score for intrusive memory strength (IMQ sum score averaged over days 0–2) to test for relationships between estradiol/progesterone and intrusive memories. Again, the potential modulating role of UCR strength or trait anxiety and depressive symptoms was explored through partial correlations.

#### 2.5.3 Mediational analyses – relationship between conditioned responding during fear extinction, intrusive memories, and ovarian hormones

Where analyses displayed a significant relationship between hormonal levels and intrusive memories, a set of additional (partial) correlational analyses assessed conditioned responding during extinction as a potential mediator. The respective analyses were conducted following the steps for mediation analysis described by Baron and Kenny (1986) and using nonparametric correlational analyses.^6^ Generally, the *α*-level for primary statistical analyses testing our main hypotheses (i.e. relationships between hormones, conditioned responding during fear extinction, and intrusive memories) was set to .05. For all other, secondary analyses, *p*-levels mainly serve descriptive purposes. Effect sizes (partial eta squared *η*^2^ or Cohen’s *d*) are reported for significant results. All statistical analyses were performed in PASW Statistics 18 (SPSS Inc., Chicago, IL, USA).

## 3 Results

### 3.1 Demographic and psychometric characteristics

Participating women’s mean age was 23.9 years (*SD* = 3.4) and their trait anxiety (*M* = 38.9, *SD* = 10.3; Laux et al., 1981) as well as depressive symptoms (*M* = 13.2, *SD* = 8.4; Hautzinger & Bailer, 1993) were in the normal range. Participants reported regular menstrual cycles with a mean cycle length of 28.9 days (*SD* = 2.9). On the day of testing, 16 participants (43.2%) were in their early follicular phase and the other 21 participants (56.8%) were in their luteal phase. All further analyses in the main text are based on continuous levels of estradiol (*M* = 6.23 pg/mL, *SD* = 3.90) and progesterone (*M* = 191.17 pg/mL, *SD* = 89.01) whereas Appendix A contains an exploratory analysis comparing women in the two cycle phases. Estradiol and progesterone levels were unrelated to age (largest *ρ*(37) = .06, smallest *p* = .719), trait anxiety (largest *r*(35) = −.17, smallest *p* = .323) or depressive symptoms (largest *r*(35) = −.11, smallest *p* = .529).

### 3.2 Physiological baseline activation and reactions to unconditioned stimuli (film clips)

Neither estradiol nor progesterone levels were significantly related to preconditioning baseline SCL (*M* = 5.28 μS, *SD* = 2.06; largest *r*(33) = .17, smallest *p* = .350; note that two participants had to be excluded from this analysis due to technical problems during baseline measurement) or electrodermal UCR (*M* = 0.90 μS, *SD* = 0.59; largest *r*(35) = −.09, smallest *p* = .619).^7^ Mean valence of film clips was 87.2 (*SD* = 11.6; 0 = very pleasant, 100 = very unpleasant) indicating that films were experienced as highly unpleasant by participants. Likewise, participants’ ratings of film clips were not significantly correlated with levels of estradiol or progesterone (largest *ρ*(37) = .14, smallest *p* = .427). Thus, neither estradiol nor progesterone levels covaried with participants’ physiological baseline activation or unconditioned film responding.

### 3.3 Fear conditioning

#### 3.3.1 Manipulation check for the fear conditioning procedure

**Fig. 1:**
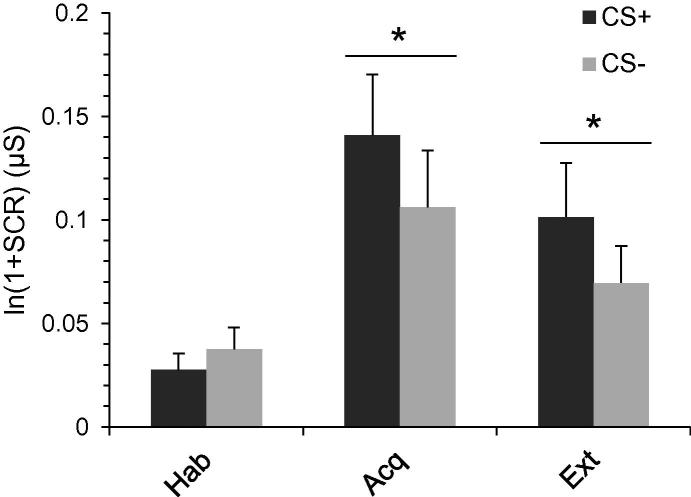
Means (and standard errors) of skin conductance responses (SCRs) to CS^+^ and CS^−^ across habituation (Hab), acquisition (Acq), and extinction (Ext) phases of the fear conditioning task. Asterisks indicate significant difference in skin conductance responses (SCR) between CS^+^ and CS^−^ trials (*p* < .05).

Participants demonstrated reliable differential electrodermal fear acquisition: The repeated-measures ANOVA for the whole conditioning task displayed a significant main effect of CS-type (*F*(1, 36) = 7.84, *p* = .008, *η*^2^ = .18) and Conditioning phase (*F*(2, 72) = 13.94, *p* < .001, *η*^2^ = .28, *ε* = .634), as well as a significant CS-type × Conditioning phase interaction (*F*(2, 72) = 4.25, *p* = .025, *η*^2^ = .11, *ε* = .828). Post-hoc tests revealed that participants’ SCRs did not significantly differ between CS^+^ and CS^−^ during habituation (*t*(36) = −1.39, *p* = .174). However, a significant difference between SCRs to CS^+^ and CS^−^ could be observed during acquisition (*t*(36) = 2.20, *p* = .035, *d* = 0.20) as well as during extinction (*t*(36) = 2.81, *p* = .008, *d* = 0.23). Thus, consistent with our previous report on the larger sample (Wegerer, Blechert, Kerschbaum, et al., 2013), these data indicate that the film-based conditioning paradigm can induce fear learning in healthy (naturally cycling) women. Fig. 1 displays means and standard errors for SCRs over the whole conditioning procedure and all participants.

#### 3.3.2 Effects of estradiol and progesterone on differential fear conditioning

**Fig. 2:**
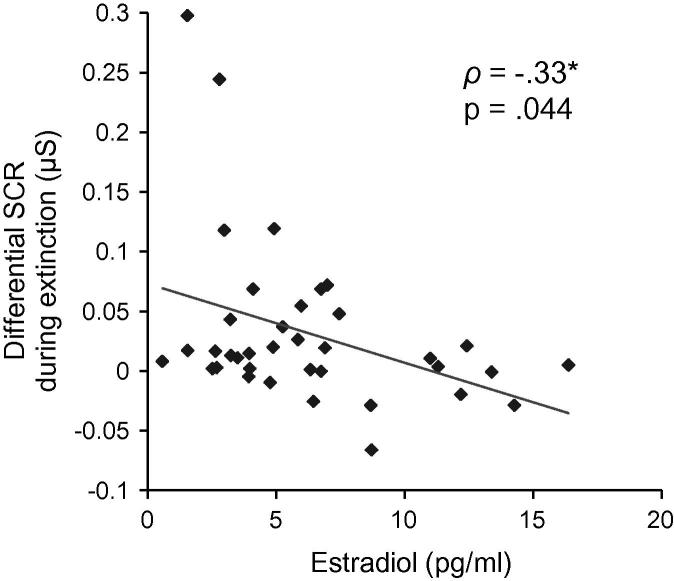
Relationship between estradiol and conditioned responding during fear extinction. See ANCOVA main effect of estradiol reported in Section 3.3.2. Differential SCR was calculated subtracting ln (1 + SCR) for CS^−^ trials from ln (1 + SCR) for CS^+^ trials. *ρ:* Spearman correlation coefficient (see Footnote 5).

Assessing estradiol and progesterone effects on differential SCRs, ANCOVA for habituation revealed neither a significant main effect of Time (*F*(1, 34) = 0.33, *p* = .569), nor a main or interaction effect involving estradiol (all *F*s(1, 34) < .0.38, *p*s > .544) or progesterone (all *F*s(1, 34) < 0.02, *p*s > .896). Similarly, during acquisition, no significant main effect of Time (*F*(1, 34) = 0.17, *p* = .683) and no main or interaction effects involving estradiol (all *F*s(1, 34) < 0.37, *p*s > .545) or progesterone (all *F*s < 0.15, *p*s > .706) were observed. During extinction, however, the main effect for estradiol was significant (*F*(1, 34) = 5.43, *p* = .026, *η*^2^ = .14), indicating a linear relationship between estradiol levels and conditioned responding during extinction in the sense that participants with lower levels of estradiol displayed larger differential responses during extinction (see Fig. 2). There was no significant main effect of Time (*F*(1, 34) = 0.93, *p* = .343) or other main or interaction effects involving estradiol (*F*(1, 34) = 0.87, *p* = .359) or progesterone (all *F*s(1, 34) < 0.14, *p*s > .713) during extinction.^8^ Including UCR to film clips, anxiety, and depressive symptoms as further control variables into ANCOVAs did not change the significant main effect of estradiol for conditioned responding during extinction (*F*(1, 31) = 4.81, *p* = .036, *η*^2^ = .13). Furthermore, other main and interaction effects involving estradiol or progesterone during habituation, acquisition or extinction remained non-significant (all *F*s(1, 31) < 0.94, *p*s > .339).

### 3.4 Ambulatory assessment of intrusive memories

**Fig. 3:**
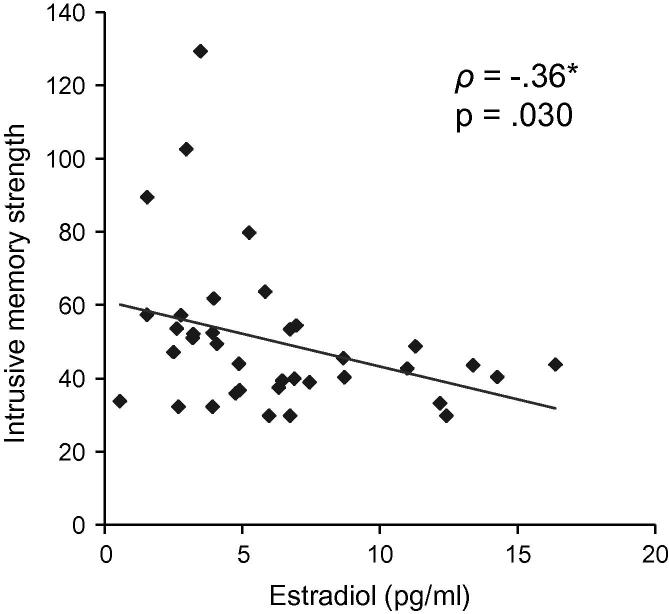
Relationship between estradiol and intrusive memory strength. Values of intrusive memory strength represent sum scores of *t*-scores of Intrusive Memory Questionnaire (IMQ) items (number, duration, and distress of intrusive memories) averaged across days 0–2. *ρ:* Spearman correlation coefficient.

Table 1 displays means and standard deviations for intrusive memories as measured by the three items of the IMQ on days 0–2 after the laboratory session. Importantly, we observed a significant negative correlation between levels of estradiol and intrusive memory strength reported on days 0–2 (*ρ*(37) = −.36, *p* = .030), indicating that women with lower levels of estradiol reported overall larger intrusive memory strength relating to the film clips used in the conditioning procedure (see Fig. 3). However, intrusive memories did not significantly correlate with progesterone (*ρ*(37) = −.08, *p* = .656). Partialling out UCR to film clips, anxiety, and depressive symptoms did not change these results much (estradiol: *ρ*(32) = −.38, *p* = .026, progesterone: *ρ*(32) = −.09, *p* = .605).

### 3.5 Mediation analyses: does conditioned responding during fear extinction mediate the association between estradiol and intrusive memories?

Estradiol was associated with differential responding during extinction (see Section 3.3.2. *above*) and with intrusive memory strength (see Section 3.4. *above*) but was the latter correlation actually mediated by individual differences in conditioned responding during extinction? In fact, further correlational analyses confirmed that conditioned responding during extinction was significantly correlated with intrusive memory strength (*ρ*(37) = .41, *p* = .012) and remained so when partialling out estradiol (*ρ*(34) = .33, *p* = .050) (*ρ* represents coefficient for partial Spearman correlation; Kendall & Gibbons, 1990). The correlation between estradiol and intrusive memory, on the other hand, fell below significance when partialling out extinction responding (*ρ*(34) = −.26, *p* = .130). Thus, results suggest that conditioned responding during fear extinction was a relevant mediating mechanism in the inverse correlation between estradiol and intrusive memory strength.^9^

## 4 Discussion

Using a novel conditioned-intrusion paradigm in naturally cycling healthy women, we observed a significant association of lower levels of estradiol with higher differential conditioned responding during extinction. Furthermore, lower levels of estradiol were significantly related to higher intrusive memory strength reported during ambulatory assessments and this effect was mediated by conditioned responding during extinction. Thus, based on our results and previous research (reviewed in Lebron-Milad et al., 2012), we conclude that low levels of estradiol promote stronger and more extinction-resistant conditioned emotional reactions – such as intrusive memories – to trauma reminders. With respect to progesterone, however, we found no significant effect on either differential fear acquisition/extinction or on intrusive memories.

Our results are consistent with a number of previous fear conditioning studies that reported a beneficial effect of estradiol on fear extinction processes. Findings slightly differ, however, in *when*, during the conditioning task, estradiol has an effect (i.e. effect on *extinction learning* on the day of acquisition vs. on *extinction recall* on the next day; also note that extinction recall was not assessed in all studies, including our own). Differences in study design (e.g. with respect to the encountered UCS, i.e. electric stimulation, air blasts, or aversive film clips) or physiological outcome variables (i.e. SCR, startle response, or functional imaging) might be one possible explanation for these different outcomes. In our study, women with lower levels of estradiol displayed higher conditioned skin conductance responses in a fear extinction session that directly followed the acquisition phase. Milad et al. (2010), however, found no significant effect of estradiol on initial extinction learning but did observe an effect on extinction recall on the next-day (see Zeidan et al., 2011, and Graham & Milad, 2013, for similar results). Yet, concordant with our results, Glover et al. (2012) found a significant effect of estradiol on fear-potentiated startle in a fear extinction session conducted on the day of acquisition. Furthermore, a positive correlation between estradiol levels and activation of the ventromedial prefrontal cortex (vmPFC) during initial extinction learning was reported by Zeidan et al. (2011). The vmPFC is known to play an essential role in fear extinction in the sense that it inhibits activity of the amygdala and other regions supporting fear responses. Concordantly, neurocircuitry models of PTSD propose an amygdala hyper-responsivity, which together with a vmPFC hypo-responsivity impairs extinction of traumatic memories (Pitman et al., 2012, Rauch et al., 2006, Sehlmeyer et al., 2009, VanElzakker et al., 2014). Another brain structure crucially involved in fear learning and extinction and showing aberrant activation in PTSD patients is the hippocampus (Pitman et al., 2012, Sehlmeyer et al., 2009, VanElzakker et al., 2014). Notably, estrogen receptors are expressed in all of these neural structures, PFC, amygdala, and hippocampus (Cui et al., 2013, Montague et al., 2008, Ostlund et al., 2003). Furthermore, studies in monkeys and rodents have demonstrated effects of estradiol on neuronal plasticity in these brain areas (Hao et al., 2006, McEwen et al., 2012, Rasia-Filho et al., 2012). Similarly, Graham and Milad (2013) attributed the extinction-enhancing effect of estradiol administration which they found in naturally cycling women to such effects of estradiol on neuronal plasticity. Importantly, the significant effect of estradiol observed during fear extinction by our and other studies is also consistent with accumulating evidence pointing to a more general beneficial role of estradiol for the regulation of anxiety (Solomon & Herman, 2009). Nevertheless, Hiroi and Neumaier (2011) cautioned that estradiol effects on anxiety-like behavior in humans and animals are rather complex and multifaceted and may stem from differential activation of different estradiol receptors in different brain pathways, mandating more systematic research.

Estradiol was also significantly negatively correlated with intrusions measured during the two days post extinction. This relationship was not attributable to estradiol effects on the perception of film clips or associated arousal (see e.g. Goldstein et al., 2005), since levels of estradiol were unrelated to both participants’ valence ratings (see also Soni et al., 2013) and electrodermal responses (UCR) to film clips. Rather, we had hypothesized an effect of estradiol on conditioned responding during extinction which would in turn facilitate subsequent triggering of intrusive memories by conditioned reminders in daily life. Such a perspective is in line with cognitive-behavioral accounts of intrusive memories as (non-extinguished) emotional reactions to trauma reminders (Ehlers and Clark, 2000, Mineka and Oehlberg, 2008). Ehlers and Clark (2000), for example, suggested that stimulus–response associations are particularly strong for traumatic material in PTSD, which would make triggering of intrusive memories by related stimuli more likely. Importantly, such a functional relationship between fear conditioning processes and intrusive memories is also supported by our previous report (Wegerer, Blechert, Kerschbaum, et al., 2013) as well as by Norrholm et al. (2011), who found conditioned responding during acquisition and extinction to be correlated with re-experiencing symptoms in patients with PTSD. Further support for this perspective comes from our mediation analysis showing that the relationship between lower estradiol and stronger intrusive memories was (at least partially) accounted for by the conditioned responding during extinction. It should be noted that although our results revealed no significant estradiol effect during acquisition, it is possible that the stronger conditioned responding during extinction in low-estradiol women was to some degree also influenced by the preceding acquisition process. Importantly, however, from the perspective of intrusive memory research, it can be assumed that stronger, non-extinguished conditioned responding is a major source of subsequent memory triggering (and therefore of primary interest for the current study), irrespective of the relative contribution of stronger initial fear acquisition vs. weaker fear extinction. Similarly, Orr et al. (2000) suggested that a combination of both acquiring a larger *and/or* more persistent autonomic differential response to an aversive CS (i.e. higher fear conditionability) might put individuals at risk of developing symptoms of PTSD.

Apart from conditioning-related explanations, other mechanisms such as hormonal influences on aversive declarative memory might have contributed to the negative correlation between estradiol and intrusive memories and need consideration. Supporting a general relationship between declarative memory strength and intrusive memories, Ferree and Cahill (2009) found a correlation between frequency of intrusive memories and the recall performance regarding details of emotional films. A possible interpretation of this finding is that stronger encoding or consolidation of emotional material might enhance memory accessibility, resulting in not only better intentional recall but also in higher intrusive memory strength. A further aspect might be declarative memory organization: As suggested by Ehlers and Clark (2000), poor elaboration and contextualization of trauma memory acts in concert with strong associative memory to promote triggering of intrusive memories. However, research linking ovarian hormones with emotional declarative memory processes is scarce and results are inconsistent (Bayer et al., 2014, Cheung et al., 2013, Ertman et al., 2011, Felmingham et al., 2012, Ferree et al., 2011, Nielsen et al., 2013). Two studies found a positive association of progesterone with recall and recognition of aversive pictures, but did not identify relationships with estradiol (Ertman et al., 2011, Felmingham et al., 2012). Of note, these authors could not rule out that the observed progesterone effects were partially due to estrogenic activity. Finally, an effect of estradiol on emotional declarative memory is at least possible from a neurobiological perspective, since estradiol modulates neuronal pathways that are known to play a central role for declarative memory encoding of emotional material (Hao et al., 2006, LaBar and Cabeza, 2006, McEwen et al., 2012, Rasia-Filho et al., 2012). In sum, a putative effect of estradiol on declarative memory strength/organization, which might furthermore influence intrusive memory strength, remains speculative to date but might be targeted by future research.

With respect to progesterone, our results are consistent with previous studies that did not find any significant effect of progesterone on fear acquisition or extinction (Milad et al., 2010, Zeidan et al., 2011). Progesterone was also unrelated to intrusive memories in our study. This finding conflicts with Ferree et al. (2011), who observed a positive relationship of progesterone with intrusive memories. The authors have suggested amygdala-mediated influences of allopregnanolone – a neuroactive metabolite of progesterone that is thought to influence anxiety-related behavior through its action on the GABA_A_ receptor – as a possible explanation for their finding (van Wingen et al., 2011). A more general role of progesterone for aversive memories is, as outlined above, furthermore supported by another line of research that found positive relationships between progesterone and deliberate (rather than spontaneous, intrusive) memory recall of aversive stimuli (Ertman et al., 2011, Felmingham et al., 2012; authors discussed effects of progesterone on the release and action of glucocorticoids as potential underlying mechanisms; see also Andreano et al., 2008, Kirschbaum et al., 1999). However, similar to our results, neither Cheung et al. (2013) nor Soni et al. (2013) found any relationship between progesterone levels and intrusive memories, thereby contributing to an inconsistent overall picture of the role of progesterone on intrusive re-experiencing. A general challenge to these studies is that influences of estradiol and progesterone can be hard to tease apart in naturally cycling women (since, for example, high progesterone levels do mainly occur in the presence of increased estradiol levels; see also Ertman et al., 2011, Milad et al., 2010). Accordingly, we cannot rule out with certainty that the effect of estradiol on intrusive memories was not in part supported by effects of progesterone. Furthermore, methodological differences might explain inconsistent study outcomes. Studies on intrusive memories differ, for example with regard to the encountered aversive material, the kind of intrusive memory assessment, inclusion criteria (e.g. with vs. without users of hormonal contraceptives, different operationalization and inclusion of menstrual cycle phases), and estimation of ovarian hormone activity (cycle phases vs. hormone assessment). We suggest that future research could benefit from a higher degree of standardization with respect to some of the aspects listed above.

### Limitations and future directions

Future studies might furthermore want to take some other possible adaptions and extensions into account. First, all of the fear conditioning studies reviewed above – the current study included – investigated influences of ovarian hormones in paradigms measuring cued fear conditioning (i.e. conditioned responding to a previously neutral CS that is paired with a UCS) rather than contextual fear conditioning (i.e. fear responding to the context in which conditioning occurred, e.g. Grillon et al., 2006, Vansteenwegen et al., 2008). Future research might explore whether the observed effects of estradiol similarly apply to context conditioning. Furthermore, our study did not assess effects of estradiol on later recall of extinction memory which might, however, constitute an additional pathway to intrusive memories: Deficits in extinction recall – such as those previously observed in women with low estradiol (Graham and Milad, 2013, Milad et al., 2010, Zeidan et al., 2011) – could also facilitate triggering of intrusive memories by conditioned trauma reminders, which could be more closely evaluated by future research. Next, intrusive memory research does mainly rely on retrospective assessment of intrusive memories even though this might be prone to memory biases. Critically, real-time assessment of intrusive memories still poses a challenge since the mere presence of assessment reminders (such as an electronic diary) easily triggers intrusive memories and could therefore artificially enhance intrusive memory reports – an issue that future developments and research might strive to overcome (see also Soni et al., 2013). Finally, it should be noted that our data were collected in a relatively small sample, thus not providing high statistical power to allow for rigid control for multiple testing (however, see Perneger, 1998, for potential pitfalls of the application of Bonferroni corrections). We tried to diminish this problem by focusing on a small set of strong empirically derived hypotheses in our primary analyses (i.e. relationships between hormones, conditioned responding during fear extinction, and intrusive memories) and supplemented these with secondary, more exploratory analyses. Furthermore, we suggest that the results of the mediational analysis should be interpreted with caution until their replication since this analysis had to be calculated non-parametrically due to non-normal bivariate distributions.

### Conclusions

Our results revealed that women with lower levels of estradiol displayed significantly higher conditioned responding during fear extinction. Furthermore, there was a significant inverse relationship between estradiol and intrusive memories, and the conditioned responding displayed during extinction (at least partially) accounted for this relationship. These results point to the possible involvement of lower estradiol levels in the susceptibility to PTSD symptoms subsequent to traumatic experiences. Finally, our results encourage further investigation of the effects of ovarian hormones on fear conditioning and intrusive memories in conjunction since doing so could provide a more conclusive picture of the role of these hormones in aversive learning and memory than studying effects in either of the two paradigms separately.

## Footnotes

1 Note that these participants were excluded to obtain a sample that could be used both for our main analyses and the exploratory analyses on menstrual cycle phases reported in Appendix A. However, retaining these four participants in the main analyses would leave the main results for estradiol and progesterone unchanged.

2 Participants who were tested in the morning vs. in the afternoon did not differ with respect to estradiol or progesterone levels (all *t*s(35) < 0.74, *p*s > .47). Furthermore, inclusion of the time of testing (either as a dichotomous variable for morning vs. afternoon or a continuous variable coding exact start time) into the main analyses did not change the significant relationship between estradiol and conditioned responding during extinction (all *F*s(1, 33) > 5.42, *p*s < .026) or intrusive memories (smallest *ρ*(34) = −.36, largest *p* = .032).

3 Habitual consumption of TV and film footage or video games depicting severe violence ranged between ‘(almost) never’ and ‘two to three times per week’ (*Mdn* = ‘a few times per year’). There were no significant relationships between participants’ violent media consumption and outcome variables including conditioned responding during acquisition and extinction or intrusive memories (largest *ρ*(37) = −.24, smallest *p* = .152) or with levels of estradiol or progesterone (largest *ρ*(37) = .18, smallest *p* *=* .283).

4 Items of the Intrusive Memory Questionnaire (IMQ): *IMQ number*: “How often did memories (in the form of images, thoughts or sounds) of the violence scenes pop into your mind today?” *IMQ duration*: “How many minutes have you roughly been engaged with memories of the violence scenes today?” *IMQ distress*: “How distressing did you find these memories (images, thoughts, or sounds)?”

5 Note that analyzing data using repeated-measures ANCOVAs (which do not have a non-parametric counterpart) can be considered accurate despite distributions of differential conditioned responses being right skewed. ANCOVA is considered quite robust to violations of multivariate normality with larger samples (as given here), and normal distribution is considered more important for the covariates, while being less crucial for the dependent variables (Mayers, 2013, Tabachnick and Fidell, 2007). Furthermore, the significant linear relationship between estradiol levels and conditioned responding during extinction as revealed by ANCOVA results is also supported by nonparametric correlational analysis (*ρ*(37) = −.33, *p* = .044; see also Fig. 2).

6 Note that our analysis does not represent an inference statistical test of mediation such as, for example, suggested by Hayes (2013; i.e. sobel test or bootstrapping to test for indirect effect). Since our data did not fulfill bivariate normal distribution and correlations were calculated based on Spearman’s *rho*, we were unable to perform such a test but conducted analyses following the steps suggested by Baron and Kenny (1986) using partial Spearman’s *rho* (Kendall & Gibbons, 1990) to test for potential mediation. However, we suggest that future studies should perform mediation analyses as described by Hayes (2013) based on larger samples that would more likely fulfill required assumptions.

7 Mean and standard deviation for UCR are reported for absolute UCR, further statistical analyses were conducted using ln (1 + UCR) to normalize data.

8 Checking for outliers, we found that one participant exceeded a *z*-score of 3.29 (Field, 2009) for differential acquisition, one participants for differential extinction, and one for intrusive memories. We checked for potential irregularities that might account for the response pattern of these participants but found no abnormalities with respect to data collection or participants’ behavior. Based on this, participants were kept in the main analyses. Nevertheless, we redid main analyses excluding participants that represented an outlier on the respective variables. When doing this, the significant main effect of estradiol on conditioned responding during extinction as well as estradiol’s negative correlation with intrusive memories remained unchanged.

9 Note that in our previous research we referenced conditioned responding during extinction to habituation levels of responding when assessing the relationship with intrusive memories (Wegerer, Blechert, Kerschbaum, et al., 2013). Aiming at simplifying our analyses, we do not additionally report results for this baseline-referenced index in the main analyses of the current report. Still, it should be noted that this previously described index does also significantly correlate with estradiol levels (*ρ*(37) = −.33, *p* = .049) as well as with intrusive memories (*ρ*(37) = .42, *p* = .011) in the current subsample.

## References

[b0005] Altshuler L.L., Hendrick V., Cohen L.S. (1998). Course of mood and anxiety disorders during pregnancy and the postpartum period. Journal of Clinical Psychiatry.

[b0010] Andreano J.M., Arjomandi H., Cahill L. (2008). Menstrual cycle modulation of the relationship between cortisol and long-term memory. Psychoneuroendocrinology.

[b0015] Baron R.M., Kenny D.A. (1986). The moderator-mediator variable distinction in social psychological research – Conceptual, strategic, and statistical considerations. Journal of Personality and Social Psychology.

[b0020] Bayer J., Schultz H., Gamer M., Sommer T. (2014). Menstrual-cycle dependent fluctuations in ovarian hormones affect emotional memory. Neurobiology of Learning and Memory.

[b0025] Bellem A., Meiyappan S., Romans S., Einstein G. (2011). Measuring estrogens and progestagens in humans: An overview of methods. Gender Medicine.

[b0030] Blechert J., Michael T., Vriends N., Margraf J., Wilhelm F.H. (2007). Fear conditioning in posttraumatic stress disorder: Evidence for delayed extinction of autonomic, experiential, and behavioural responses. Behaviour Research and Therapy.

[b0035] Boucsein W. (2012).

[b0040] Boucsein W., Fowles D.C., Grimnes S., Ben-Shakhar G., Roth W.T., Dawson M.E. (2012). Publication recommendations for electrodermal measurements. Psychophysiology.

[b0045] Brinton R.D., Thompson R.F., Foy M.R., Baudry M., Wang J., Finch C.E. (2008). Progesterone receptors: Form and function in brain. Frontiers in Neuroendocrinology.

[b0050] Bryant R.A., Felmingham K.L., Silove D., Creamer M., O’Donnell M., McFarlane A.C. (2011). The association between menstrual cycle and traumatic memories. Journal of Affective Disorders.

[b0055] Cheung J., Chervonsky L., Felmingham K.L., Bryant R.A. (2013). The role of estrogen in intrusive memories. Neurobiology of Learning and Memory.

[b0060] Cui J., Shen Y., Li R. (2013). Estrogen synthesis and signaling pathways during aging: from periphery to brain. Trends in Molecular Medicine.

[b0065] Ehlers A., Clark D.M. (2000). A cognitive model of posttraumatic stress disorder. Behaviour Research and Therapy.

[b0070] Ehring T., Fuchs N., Kläsener I. (2009). The effects of experimentally induced rumination versus distraction on analogue posttraumatic stress symptoms. Behavior Therapy.

[b0075] Ertman N., Andreano J.M., Cahill L. (2011). Progesterone at encoding predicts subsequent emotional memory. Learning and Memory.

[b0080] Farage M.A., Osborn T.W., MacLean A.B. (2008). Cognitive, sensory, and emotional changes associated with the menstrual cycle: A review. Archives of Gynecology and Obstetrics.

[b0085] Felmingham K.L., Fong W.C., Bryant R.A. (2012). The impact of progesterone on memory consolidation of threatening images in women. Psychoneuroendocrinology.

[b0090] Ferree N.K., Cahill L. (2009). Post-event spontaneous intrusive recollections and strength of memory for emotional events in men and women. Consciousness and Cognition.

[b0095] Ferree N.K., Kamat R., Cahill L. (2011). Influences of menstrual cycle position and sex hormone levels on spontaneous intrusive recollections following emotional stimuli. Consciousness and Cognition.

[b0100] Field A. (2009).

[b0105] Foa E.B., Zinbarg R.E., Rothbaum B.O. (1992). Uncontrollability and unpredictability in post-traumatic stress disorder: An animal model. Psychological Bulletin.

[b0110] Glover E.M., Jovanovic T., Mercer K.B., Kerley K., Bradley B., Ressler K.J. (2012). Estrogen levels are associated with extinction deficits in women with posttraumatic stress disorder. Biological Psychiatry.

[b0115] Glover E.M., Mercer K.B., Norrholm S.D., Davis M., Duncan E., Bradley B. (2013). Inhibition of fear is differentially associated with cycling estrogen levels in women. Journal of Psychiatry and Neuroscience.

[b0120] Goldstein J.M., Jerram M., Poldrack R., Ahern T., Kennedy D.N., Seidman L.J. (2005). Hormonal cycle modulates arousal circuitry in women using functional magnetic resonance imaging. Journal of Neuroscience.

[b0125] Graham B.M., Milad M.R. (2013). Blockade of estrogen by hormonal contraceptives impairs fear extinction in female rats and women. Biological Psychiatry.

[b0130] Grillon C., Baas J.M., Cornwell B., Johnson L. (2006). Context conditioning and behavioral avoidance in a virtual reality environment: Effect of predictability. Biological Psychiatry.

[b0135] Hao J., Rapp P.R., Leffler A.E., Leffler S.R., Janssen W.G., Lou W. (2006). Estrogen alters spine number and morphology in prefrontal cortex of aged female rhesus monkeys. Journal of Neuroscience.

[b0140] Hautzinger M., Bailer M. (1993).

[b0145] Hayes A.F. (2013).

[b0150] Hiroi R., Neumaier J.F. (2011). Complex roles of estrogen in emotion: sex matters. Biological Psychiatry.

[b0155] Kendall M.G., Gibbons J.D. (1990).

[b0160] Kirschbaum C., Kudielka B.M., Gaab J., Schommer N.C., Hellhammer D.H. (1999). Impact of gender, menstrual cycle phase, and oral contraceptives on the activity of the hypothalamus-pituitary adrenal axis. Psychosomatic Medicine.

[b0165] LaBar K.S., Cabeza R. (2006). Cognitive neuroscience of emotional memory. Nature Reviews Neuroscience.

[b0170] Laux L., Glanzmann P., Schaffner P., Spielberger C.D. (1981).

[b0175] Lebron-Milad K., Graham B.M., Milad M.R. (2012). Low estradiol levels: A vulnerability factor for the development of posttraumatic stress disorder. Biological Psychiatry.

[b0180] Lebron-Milad K., Milad M.R. (2012). Sex differences, gonadal hormones and the fear extinction network: Implications for anxiety disorders. Biology of Mood & Anxiety Disorders.

[b0185] Lommen M.J., Engelhard I.M., Sijbrandij M., van den Hout M.A., Hermans D. (2013). Pre-trauma individual differences in extinction learning predict posttraumatic stress. Behaviour Research and Therapy.

[b0190] Mayers A. (2013).

[b0195] McEwen B.S., Akama K.T., Spencer-Segal J.L., Milner T.A., Waters E.M. (2012). Estrogen effects on the brain: Actions beyond the hypothalamus via novel mechanisms. Behavioral Neuroscience.

[b0200] Michael T., Blechert J., Vriends N., Margraf J., Wilhelm F.H. (2007). Fear conditioning in panic disorder: Enhanced resistance to extinction. Journal of Abnormal Psychology.

[b0205] Milad M.R., Goldstein J.M., Orr S.P., Wedig M.M., Klibanski A., Pitman R.K. (2006). Fear conditioning and extinction: Influence of sex and menstrual cycle in healthy humans. Behavioral Neuroscience.

[b0210] Milad M.R., Zeidan M.A., Contero A., Pitman R.K., Klibanski A., Rauch S.L. (2010). The influence of gonadal hormones on conditioned fear extinction in healthy humans. Neuroscience.

[b0215] Mineka S., Oehlberg K. (2008). The relevance of recent developments in classical conditioning to understanding the etiology and maintenance of anxiety disorders. Acta Psychologica (Amst).

[b0220] Montague D., Weickert C.S., Tomaskovic-Crook E., Rothmond D.A., Kleinman J.E., Rubinow D.R. (2008). Oestrogen receptor alpha localisation in the prefrontal cortex of three mammalian species. Journal of Neuroendocrinology.

[b0225] Moses-Kolko E.L., Berga S.L., Kalro B., Sit D.K.Y., Wisner K.L. (2009). Transdermal estradiol for postpartum depression: A promising treatment option. Clinical Obstetrics and Gynecology.

[b0230] Nielsen S.E., Ahmed I., Cahill L. (2013). Sex and menstrual cycle phase at encoding influence emotional memory for gist and detail. Neurobiology of Learning and Memory.

[b0235] Norrholm S.D., Jovanovic T., Olin I.W., Sands L.A., Karapanou I., Bradley B. (2011). Fear extinction in traumatized civilians with posttraumatic stress disorder: Relation to symptom severity. Biological Psychiatry.

[b0240] Olff M., Langeland W., Draijer N., Gersons B.P.R. (2007). Gender differences in posttraumatic stress disorder. Psychological Bulletin.

[b0245] Orr S.P., Metzger L.J., Lasko N.B., Macklin M.L., Peri T., Pitman R.K. (2000). De novo conditioning in trauma-exposed individuals with and without posttraumatic stress disorder. Journal of Abnormal Psychology.

[b0250] Ostlund H., Keller E., Hurd Y.L. (2003). Estrogen receptor gene expression in relation to neuropsychiatric disorders. Annals of the New York Academy of Sciences.

[b0255] Peri T., Ben-Shakhar G., Orr S.P., Shalev A.Y. (2000). Psychophysiologic assessment of aversive conditioning in posttraumatic stress disorder. Biological Psychiatry.

[b0260] Perneger T.V. (1998). What’s wrong with Bonferroni adjustments. BMJ.

[b0265] Pitman R.K., Rasmusson A.M., Koenen K.C., Shin L.M., Orr S.P., Gilbertson M.W. (2012). Biological studies of post-traumatic stress disorder. Nature Reviews Neuroscience.

[b0270] Rasia-Filho A.A., Dalpian F., Menezes I.C., Brusco J., Moreira J.E., Cohen R.S. (2012). Dendritic spines of the medial amygdala: Plasticity, density, shape, and subcellular modulation by sex steroids. Histology and Histopathology.

[b0275] Rauch S.L., Shin L.M., Phelps E.A. (2006). Neurocircuitry models of posttraumatic stress disorder and extinction: Human neuroimaging research – Past, present, and future. Biological Psychiatry.

[b0280] Sacher J., Okon-Singer H., Villringer A. (2013). Evidence from neuroimaging for the role of the menstrual cycle in the interplay of emotion and cognition. Frontiers in Human Neuroscience.

[b0285] Schmidt P.J., Rubinow D.R. (2009). Sex hormones and mood in the perimenopause. Annals of the New York Academy of Sciences.

[b0290] Sehlmeyer C., Schoning S., Zwitserlood P., Pfleiderer B., Kircher T., Arolt V. (2009). Human fear conditioning and extinction in neuroimaging: A systematic review. PLoS ONE.

[b0295] Shirtcliff E.A., Granger D.A., Schwartz E., Curran M.J. (2001). Use of salivary biomarkers in biobehavioral research: Cotton-based sample collection methods can interfere with salivary immunoassay results. Psychoneuroendocrinology.

[b0300] Solomon M.B., Herman J.P. (2009). Sex differences in psychopathology: Of gonads, adrenals and mental illness. Physiology & Behavior.

[b0305] Soni M., Curran V.H., Kamboj S.K. (2013). Identification of a narrow post-ovulatory window of vulnerability to distressing involuntary memories in healthy women. Neurobiology of Learning and Memory.

[b0310] Tabachnick G.G., Fidell L.S. (2007).

[b0315] Tolin D.F., Foa E.B. (2006). Sex differences in trauma and posttraumatic stress disorder: A quantitative review of 25 years of research. Psychological Bulletin.

[b0320] van Wingen G.A., Ossewaarde L., Bäckström T., Hermans E.J., Fernández G. (2011). Gonadal hormone regulation of the emotion circuitry in humans. Neuroscience.

[b0325] VanElzakker M.B., Dahlgren M.K., Davis F.C., Dubois S., Shin L.M. (2014). From Pavlov to PTSD: The extinction of conditioned fear in rodents, humans, and anxiety disorders. Neurobiology of Learning and Memory.

[b0330] Vansteenwegen D., Iberico C., Vervliet B., Marescau V., Hermans D. (2008). Contextual fear induced by unpredictability in a human fear conditioning preparation is related to the chronic expectation of a threatening US. Biological Psychology.

[b0335] Wegerer M., Blechert J., Kerschbaum H., Wilhelm F.H. (2013). Relationship between fear conditionability and aversive memories: Evidence from a novel conditioned-intrusion paradigm. PLoS ONE.

[b0340] Wegerer M., Blechert J., Wilhelm F.H. (2013). Emotionales Lernen: Ein naturalistisches experimentelles Paradigma zur Untersuchung von Angsterwerb und Extinktion mittels aversiver Filme. [Emotional learning: A naturalistic experimental paradigm for investigating fear acquisition and extinction using aversive films]. Zeitschrift für Psychiatrie, Psychologie und Psychotherapie.

[b0345] Wessa M., Flor H. (2007). Failure of extinction of fear responses in posttraumatic stress disorder: Evidence from second-order conditioning. American Journal of Psychiatry.

[b0350] Wilhelm, F. H., & Peyk, P. (2005). ANSLAB: Autonomic Nervous System Laboratory (Version 4.0). Society for Psychophysiological Research website. <http://www.sprweb.org> Accessed 03.05.12.

[b0355] Wilhelm F.H., Grossman P. (2010). Emotions beyond the laboratory: Theoretical fundaments, study design, and analytic strategies for advanced ambulatory assessment. Biological Psychology.

[b0360] Zeidan M.A., Igoe S.A., Linnman C., Vitalo A., Levine J.B., Klibanski A. (2011). Estradiol modulates medial prefrontal cortex and amygdala activity during fear extinction in women and female rats. Biological Psychiatry.

[b0365] Zetsche U., Ehring T., Ehlers A. (2009). The effects of rumination on mood and intrusive memories after exposure to traumatic material: An experimental study. Journal of Behavior Therapy and Experimental Psychiatry.

