# Supplementary material for: Low levels of estradiol are associated with elevated conditioned responding during fear extinction and with intrusive memories in daily life
Source: Neurobiol Learn Mem. 2014 Dec;116:145–54. doi: 10.1016/j.nlm.2014.10.001 (PMC4256064; doi:10.1016/j.nlm.2014.10.001)
Supplement: Supplementary data 1 [file mmc1.docx]

***Appendix A***

**Results: Influences of menstrual cycle phase**

*Endocrinologic, demographic, and psychometric characteristics*

Progesterone levels were significantly higher in women who were assessed during their luteal phase (*p*=.002; see *Table A1* for descriptive and inference statistics for all endocrinologic, demographic, and psychometric variables as well as for physiological baseline activity and reactions to film clips). However, the two groups did not significantly differ with respect to their estradiol levels (*p*=.744). Furthermore women during early follicular or luteal phase did not significantly differ in age, trait anxiety, or depressive symptoms (all *p*s>.242).

*Physiological baseline activation and reactions to unconditioned stimuli (film clips)*

Women during early follicular or luteal phase did not significantly differ with respect to their baseline SCL, mean UCR as assessed by skin conductance, or film ratings (all *p*s>.412).

*Table A1. Descriptive and inference statistics for women during early follicular vs. luteal cycle phase*

|  | **Early follicular phase** (N=16) | **Luteal phase**  (N=21) |  |
| --- | --- | --- | --- |
|  | *M (SD)* | *M (SD)* | *t,* *p*, *d* ^1^ |
| **Endocrinologic, demographic and psychometric characteristics** | | | |
|  |  |  |  |
| estradiol (in pg/ml) | 5.99 (3.65) | 6.42 (4.15) | -0.33, .744 |
| progesterone (in pg/ml) | 142.15 (53.44) | 228.53 (93.45) | -3.30, .002*, -1,13 |
| age | 24.63 (4.13) | 23.29 (2.70) | 1.19, .242 |
| trait anxiety (STAI-T) ^2^ | 40.19 (12.11) | 37.86 (8.93) | 0.68, .504 |
| depressive symptoms (ADS-L) ^3^ | 12.00 (8.76) | 14.10 (8.15) | -0.75, .458 |
| **Physiological baseline activation and reactions to unconditioned stimuli** | | | |
|  |  |  |  |
| baseline SCL (in μS) | 5.49 (2.64) | 5.11 (1.47) | 0.54, .591 |
| mean UCR (in μS) ^4^ | 0.84 (0.64) | 0.95 (0.56) | -0.81, .421 |
| film rating | 85.38 (12.63) | 88.59 (10.85) | -0.83, .412 |

*Note:* *significant at p<.05

^1^ *df*=35 for all variables, except baseline SCL (df=33; due to technical problems during measurement of baseline SCL in two participants

^2^ State-Trait Anxiety Inventory, German version by Laux, Glanzmann, Schaffner, and Spielberger (1981)

^3^ General Depression Scale, German version by Hautzinger and Bailer (1993)

^4^ means are given for absolute UCR in μS, *t*-test was calculated based on natural logarithm-transformed mean UCR (ln(1+UCR) in μS) to normalize data

*Fear conditioning*

Influences of menstrual cycle phase on fear conditioning were tested using repeated-measures ANOVAs for each conditioning phase (habituation, acquisition, extinction) with differential SCRs as an outcome variable, Time (first and second block of each phase of the respective conditioning phase) as a within-subject factor and Cycle phase as a between-subject factor. During habituation, we found neither a main nor an interaction effect involving Cycle phase (all *F*s(1,35)< 0.62, *p*>.436). However, during acquisition we observed a significant main effect of Cycle phase (*F*(1,35)=9.15, *p*=.005, *η^2^*=.21) (but no interaction of Cycle phase with Time, *F*(1,35)=0.22, *p*=.646) indicating that women in the luteal phase displayed greater differential SCRs during acquisition than women in the early follicular phase. During extinction, there was no significant main or interaction effect involving Cycle phase (all *F*s(1,35)<0.26, *p*>.615).

*Ambulatory assessment of intrusive memories*

Women in the early follicular and luteal phase did not significantly differ with respect to intrusive memory strength that they reported on day 0-2 after the laboratory study (early follicular: *M*=51.0, *SD*=16.6; luteal: *M*=49.2, *SD*=24.0; *t*(35)=0.25, *p*=.804).^1^

**Discussion: Influences of menstrual cycle phase**

As expected (Farage, Neill, & MacLean, 2009), women during the luteal phase displayed significantly higher levels of progesterone than women during early follicular phase. However, we found no significant difference between cycle phases with respect to estradiol concentrations (even though estradiol levels were higher during luteal phase at least on the descriptive level; also note that there were no outliers with respect to estradiol or progesterone levels within the two cycle phases). One possible explanation for this result could be the fact that we used a between-subject design to compare women during different cycle phases, since participants can hardly be meaningfully retested in a fear conditioning experiment. The menstrual cycle can be highly variable even within similarly aged women (Mihm, Gangooly, & Muttukrishna, 2011). Thus, testing women in a between-subject design could add between-subject variance, which might result in a mean hormonal pattern that could slightly diverge from a pattern that would be expected based on a stereotypic individual menstrual cycle. Furthermore, self-reports on menstrual cycle phase can be inaccurate (however, note that we did not only assess the day of women’s last menses but also the first day of their next menses in follow-up) and hormonal concentrations tend to overlap between the cycle phases rather than clearly differentiating between them (e.g., Ferree, Kamat, & Cahill, 2011). There are also other studies that did not observe a significant difference in estradiol concentrations between women in the early follicular and luteal phase (e.g., Andreano & Cahill, 2010; Merz et al., 2012).

With respect to fear acquisition/extinction and intrusive memories, the only difference we observed between cycle phases was that women in the luteal phase displayed significantly higher differential SCRs during acquisition than women in the follicular phase. Since the two cycle phases did not significantly differ with respect to estradiol concentrations and our main results did not reveal an effect of progesterone (or estradiol) on fear acquisition, this result is hardly explained by mere effects of estradiol or progesterone. Furthermore, we found no effect of the estradiol-to-progesterone ratio on differential conditioned responses during acquisition (results not shown above). Importantly, however, different menstrual cycle phases are not only characterized by different levels of estradiol and progesterone, but constitute changes in complex hormonal milieus that are accompanied by extensive physiological changes (Farage et al., 2009; Farage, Osborn, & MacLean, 2008). However, based on our data we are not able to unravel which factor(s) caused the difference in differential fear acquisition between the early follicular and luteal phases.

Investigating effects of different hormonal states on neuronal activation during fear acquisition, Merz et al. (2012) found no difference between women in the early follicular and luteal phase. To the best of our knowledge, there is no other human fear conditioning study that explicitly compared women during the early follicular and the luteal phase with respect to fear acquisition. In an early study, van der Molen, Merckelbach, and van den Hout (1988), found stronger fear acquisition in women during the late luteal (premenstrual) phase as compared to women in other cycle phases. However, the authors did not discriminate between women in the early follicular, late follicular, or the early luteal phase. In still another study, Merz, Stark, Vaitl, Tabbert, and Wolf (2013) reported that women during the luteal phase displayed higher conditioned-responding in the amygdala than women taking oral contraceptives (resulting in low levels of endogenous estradiol and progesterone) in an instructed fear conditioning paradigm. However, this study did not investigate women in the early follicular phase. Thus, it remains to be seen whether future studies will replicate the finding of stronger differential conditioned responses during fear acquisition in women during the luteal as compared to the early follicular phase and which factor(s) might account for this effect.

**Footnotes**

1 All results remained the same when excluding participants that represented outliers (z-score >3.29 or <-3.29; Field, 2009) on differential acquisition, extinction, or intrusive memories from the respective analyses.

**References**

Andreano, J. M., & Cahill, L. (2010). Menstrual cycle modulation of medial temporal activity evoked by negative emotion. *Neuroimage, 53*, 1286-1293.

Farage, M. A., Neill, S., & MacLean, A. B. (2009). Physiological changes associated with the menstrual cycle. A review. *Obstet Gynecol Surv, 64*(1), 58-72.

Farage, M. A., Osborn, T. W., & MacLean, A. B. (2008). Cognitive, sensory, and emotional changes associated with the menstrual cycle: a review. *Arch Gynecol Obstet, 278*(4), 299-307.

Ferree, N. K., Kamat, R., & Cahill, L. (2011). Influences of menstrual cycle position and sex hormone levels on spontaneous intrusive recollections following emotional stimuli. *Conscious Cogn, 20*(4), 1154-1162.

Field, A. (2009). *Discovering statistics using SPSS. 3rd edition*. London: SAGE Publications.

Hautzinger, M., & Bailer, M. (1993). *Allgemeine Depressionsskala [General Depression Scale]*. Göttingen: Beltz Test GmBH.

Laux, L., Glanzmann, P., Schaffner, P., & Spielberger, C. D. (1981). *Das State-Trait-Angstinventar. Theoretische Grundlagen und Handanweisung. [The State-Trait Anxiety Inventory. Theoretical basics and manual.]*. Weinheim: Beltz Test GmBH.

Merz, C. J., Stark, R., Vaitl, D., Tabbert, K., & Wolf, O. T. (2013). Stress hormones are associated with the neuronal correlates of instructed fear conditioning. *Biol Psychol, 92*(1), 82-89.

Merz, C. J., Tabbert, K., Schweckendiek, J., Klucken, T., Vaitl, D., Stark, R., & Wolf, O. T. (2012). Oral contraceptive usage alters the effects of cortisol on implicit fear learning. *Horm Behav, 62*(4), 531-538.

Mihm, M., Gangooly, S., & Muttukrishna, S. (2011). The normal menstrual cyle in women. *Anim Reprod Sci, 124*, 229-236.

van der Molen, G. M., Merckelbach, H., & van den Hout, M. A. (1988). The possible relation of the menstrual cycle to susceptibility to fear acquisition. *J Behav Ther Exp Psychiatry, 19*(2), 127-133.
